# Unravelling the true biopsychosocial impact of schistosomiasis

**DOI:** 10.1016/j.pt.2026.03.011

**Published:** 2026-05

**Authors:** Derick N.M. Osakunor, Sergi Alonso, Sandra Jumbe, Poppy H.L. Lamberton

**Affiliations:** 1 School of Biodiversity, One Health and Veterinary Medicine, University of Glasgow, Glasgow, UK; 2 Tackling Infections to Benefit Africa (TIBA) Partnership, University of Edinburgh, Edinburgh, UK; 3 Global Business School for Health, University College London, London, UK; 4 School of Social and Health Sciences, Millennium University, Blantyre, Malawi; 5 Centre for Evaluation and Methods, Wolfson Institute of Population Health, Barts, Queen Mary University of London, London, UK

**Keywords:** biopsychosocial, economic burden, health-related quality of life, impact, physical, *Schistosoma*

## Abstract

Schistosomiasis remains a debilitating parasitic disease, with over 200 million people infected globally. Heavy, chronic infections cause severe morbidity and over 12 000 deaths a year. As diagnostics improve, we have a greater understanding of more accurate prevalences. However, how infections, including light-intensity infections, affect individuals’ health and quality of life remains to be fully understood, particularly as specific markers of morbidity, disease progression, and resolution are lacking. We explore evidence about the true broader physical, psychological, economic, social, and environmental impacts of schistosomiasis, identifying critical gaps in understanding the interplay between infection, morbidity, and wellbeing, and propose innovative approaches to better capture true long-term disease impacts. We argue for more holistic frameworks to inform disease monitoring, intervention evaluation, and policy development.

## Highlights

Schistosomiasis imposes a substantial but systematically underestimated long-term physical, psychological, and socio-economic burden that extends beyond traditional disease metrics.
Delayed response between infection, morbidity, recovery trajectory, and health-related quality of life, as well as the lack of improved diagnostics for infection and morbidity, and surveillance tools, complicates burden estimation and intervention impact monitoring.
Commonly used generic health-related quality of life instruments exhibit strong ceiling effects in endemic populations, likely due to habituation to chronic ill health, inferring methodological limitations in current burden and intervention benefit assessments.
Advancing strategic control and policy requires refined metrics of infection, morbidity, health-related quality of life, and lived experiences that better capture the true biopsychosocial impact of schistosomiasis and guide more effective elimination efforts.

## Schistosomiasis: beyond the obvious

Human **schistosomiasis** (see Glossary) poses a significant global health challenge in impoverished tropical and subtropical regions of Africa, Asia, the Caribbean, and South America [1]. The disease affects over 200 million people worldwide, nearly 90% of whom live in sub-Saharan Africa, and an estimated 779 million remain at risk 1, 2. Repeated **mass drug administration (MDA)** with the anthelmintic praziquantel is the main World Health Organization (WHO)-recommended control strategy to reduce infection intensity, morbidity, and transmission, implemented through national control programmes 2, 3. Complementary interventions such as improvements in water, sanitation, and hygiene (WASH) infrastructures; access; and their use can further reduce transmission and infections, but these tend to be limited by cost, infrastructure, and implementation challenges. However, WASH improvements may be more cost-effective over longer time horizons by helping to reduce transmission, infections, and prevent morbidity. Despite decades of control efforts, schistosomiasis continues to impose a substantial burden on individuals and communities, extending beyond its well-recognised clinical manifestations.

Although the health implications of schistosomiasis are well documented [3], significant gaps persist in understanding morbidity associated with light-intensity infections, including cases where individuals are antigen-positive but egg-negative, as well as issues with time lags from infection to morbidity. A key challenge is that infection intensity is often used as a proxy for morbidity, but the associations are complex, and current infections can lead to chronic disease with long-term health impacts beyond immediate clinical manifestations. Emerging controlled human infection studies are beginning to address some knowledge gaps [4], and two other key areas remain an evolving area of study: the broader impact of infection on **health-related quality of life (HRQoL)** and the spillover effects of the costs associated with infection on households and communities, which can accrue to national levels. Moreover, the growing awareness of the inseparable nature of physical, psychological, and social factors of illness and health outcomes has led to a shift from biomedical models of care towards a biopsychosocial framework for research and intervention [5]. This approach promotes holistic, patient-centred assessment beyond HRQoL to capture the full spectrum of how these factors interact to influence disease processes and a person’s wellbeing [5]. This will include the impact of schistosomiasis ranging from physical and mental health to broader aspects of wellbeing such as educational attainment, labour productivity, and economic opportunities, all of which contribute to downstream economic burden perpetuating the cycle of poverty 6, 7. However, the lack of adequate evidence on the true individual and societal impact of schistosomiasis limits our ability to accurately evaluate intervention cost-effectiveness,—something which is essential for prioritising interventions requiring substantial upfront investment for successful long-term control and elimination, helping to identify which ‘best buys’ should be supported for long-term goals.

In this review, we (i) examine the multifaceted impact of schistosomiasis, including its physical, psychological, economic, social, and environmental dimensions; (ii) highlight critical gaps in understanding the complex interplay between schistosome infection, morbidity, health, and wellbeing; and (iii) advocate for innovative, interdisciplinary research priorities to better understand the effects of schistosomiasis on health and wellbeing.

## Multifaceted and long-term impact of schistosomiasis

**Figure 1:**
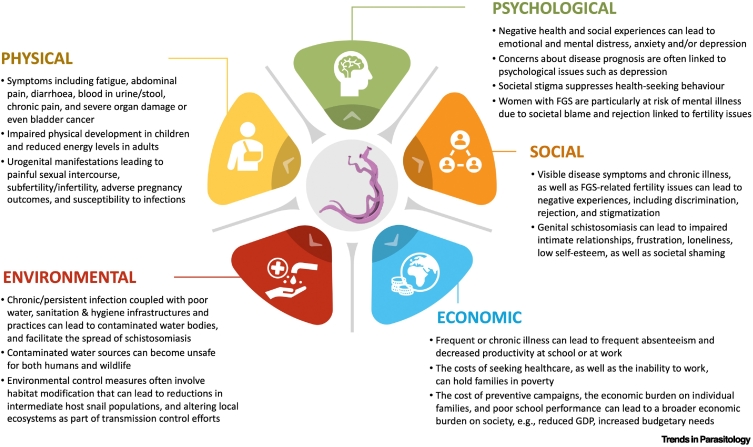
Multifaceted impact of schistosomiasis: Examples of the potential multifaceted physical, psychological, social, economic, and environmental consequences, including spillover impacts, of schistosomiasis. GDP: gross domestic product; FGS: female genital schistosomiasis.

The impact of schistosomiasis extends far beyond infection status, affecting physical and psychological health, social and economic functioning, and the environment people live in across the life course (Figure 1). Although traditionally characterised as a disease of childhood, schistosomiasis often results in chronic morbidity that persists into adolescence and adulthood, particularly in settings of sustained exposure and repeated reinfection 3, 7.

### Physical health

Individuals with schistosomiasis can experience a range of mild to debilitating symptoms, including fatigue, abdominal pain, diarrhoea, and blood in urine or stool, depending on the species involved. These early manifestations reflect acute immune responses and tissue damage at sites of egg deposition [3]. As infections become chronic, eggs trapped in host tissues trigger granulomatous inflammation and fibrosis, which can lead to long-term complications such as anaemia, hepatosplenomegaly, malnutrition, stunted growth, ureteral obstruction, irreversible bladder and kidney damage, and an elevated risk of cancer, particularly bladder cancer associated with *Schistosoma haematobium* [3]. An often under-recognised but critical component of the physical burden of schistosomiasis is when it involves the genital tract. **Female and male genital schistosomiasis (FGS/MGS)** can cause persistent reproductive tract pathology (including genital lesions, fibrosis, and dyspareunia), painful intercourse, sexual dysfunction, infertility, adverse pregnancy outcomes, and increased susceptibility to sexually transmitted infections, including HIV [8].

Whilst heavier chronic infections generally lead to more severe disease, associations between infection intensity and morbidity are not linear and are inconsistent across species and settings (Box 1) 9, 10, 11. Importantly, accumulating evidence challenges the notion of ‘asymptomatic’ schistosomiasis: individuals with light to moderate infections may still experience clinically significant morbidity 11, 24, 25, yet remain largely invisible to surveillance systems and treatment thresholds. Whilst improvements are being made in the diagnosis of *Schistosoma* infections, there is a need for improved morbidity markers that accurately measure morbidity and its resolution.

Box 1 **Evidence of schistosomiasis impact across settings**

**Infection intensity and physical morbidity.**

• Studies have demonstrated a positive correlation between egg burden and classic markers of morbidity including blood in urine and stool 9, 10, 11. However, these relationships are inconsistent across *Schistosoma* species and settings; for example, a recent study of school-aged children in six African countries found correlations between infection intensity and morbidity for *S. haematobium* but not for *S. mansoni*, which may reflect both methodological limitations (such as omission of certain ultrasound indicators), and biological differences in disease presentations across *Schistosoma* species and potentially greater overlap of symptoms with other coinfections [11].

**Psychological and social impacts.**

• Evidence on mental health outcomes remains sparse. For instance, a systematic review of NTD-related mental health found no schistosomiasis-specific studies [12]. This may reflect overreliance on weaker, subjective self-reported measures [13], prioritisation of physical health under the medical model over biopsychosocial approaches [14] or lack of qualitative studies on people’s experiences of mental illness associated with all disease stages. Data suggest mental distress may be linked with advanced disease; for example, 62% of patients with advanced *S. japonicum* reported depressive symptoms [15].
• Misconceptions about causes and transmission (e.g., witchcraft and sexual transmission) contribute to stigma, with women affected by FGS being particularly vulnerable to blame for infertility and social rejection 16, 17, 18.
• Most research focuses on cognitive impacts (e.g., reduced school attendance, memory, and achievement) [19]; however, emotional outcomes are rarely assessed. Limited evidence suggests that schistosome infection is associated with poorer emotional/behavioural wellbeing in children. In Tanzania, 6.5% of children with *S. mansoni* infection reported moderate to severe anxiety/depression [20], and in Kenya, treatment improved anxiety, depressive symptoms, hyperactivity, and school-related difficulties [21].

**Economic burden and spillover effects.**

• Empirical analyses have linked chronic schistosomiasis to reduced labour productivity and earnings, particularly in physically demanding occupations, with implications for household income and economic resilience 6, 22. Additionally, schistosomiasis-related morbidity generates indirect costs at household and community levels, including caregiving demands, forgone education, and reduced economic participation, which may aggregate to constrain local and national development. Although precise estimates are scarce, available data indicate substantial economic losses. In Burkina Faso, schistosomiasis is estimated to reduce gross domestic product by 0.8% annually through agricultural impacts, with potential crop yield gains of 7–32% if eliminated [23].

### Psychological health

Whilst the physical manifestations of schistosomiasis are well recognised, its impacts on psychological health (emotional, mental, and social wellbeing, beyond the mere absence of mental illness) remain underexplored. With up to 80% of people in low- to middle-income countries likely to experience a mental disorder in their lifetime [26], this compounds the psychological burden of **neglected tropical diseases (NTDs)** such as schistosomiasis.

Evidence from other NTDs suggests substantial burdens of depression, anxiety, and stress, yet studies examining the association between schistosomiasis and mental health are limited [27]. Like many NTDs, schistosomiasis can be linked to negative social experiences such as discrimination, rejection, and stigma, which can increase the risk of mental health problems [28]. In addition, long-term fatigue and feeling unwell can directly lower mood [15]. Being unable to attend school, interact with peers, or maintain employment due to illness and loss of income can further compound one’s psychological burden [19].

The small body of existing evidence suggests that mental health issues seem very much tied to advanced stages of schistosomiasis [15], particularly due to concerns about prognosis and long-term complications 29, 30, 31. Women affected by FGS and school-aged children may be especially vulnerable, facing compounded risks from stigma and poor emotional wellbeing 16, 20. The limited use of validated mental health instruments and overreliance on cross-sectional designs continue to obscure the full psychological burden of schistosomiasis (Box 1).

### Economic, social, and environmental effects

The economic burden of schistosomiasis extends well beyond direct medical expenses, encompassing a range of impacts (Box 1). This burden is estimated using the concept of opportunity cost [32]: the extent of the economic burden of disease, accounting for both direct (e.g., medical and transport costs) and indirect costs (e.g., time lost travelling for medical care or due to physical or psychological illness, school/labour absenteeism, and productivity losses 6, 33). The cognitive deficits and poor school performance associated with chronic schistosomiasis can also translate into long-term economic consequences, including decreased productivity and reduced employment opportunities 6, 19, 22.

At the community and sector level, schistosomiasis disrupts water-dependent livelihoods such as farming and fishing, whilst zoonotic transmission imposes additional costs on livestock health and agricultural output 19, 22. Although MDA remains relatively inexpensive (average US$0.06–US$4.46 per person [34]), incomplete coverage and the need for repeated delivery to large at-risk populations generate sustained financial demands for governments [34]. Furthermore, there is still not enough drug to treat all those in need, let alone their infected livestock, posing a major challenge to control and elimination strategies.

### Spillover effects

Overall, the impact of schistosomiasis extends beyond individual health, with significant spillover effects that impact not only the infected individuals but also their families, communities, healthcare providers, and even national systems, reinforcing cycles of poverty. The high cost of disease control and financial strains on infected individuals, including reduced productivity that limits future wages and employment opportunities [35] accrue over time, reinforcing cycles of ill-health and limiting economic growth and development in endemic regions [36]. Individuals in endemic areas remain vulnerable to infection due to inadequate WASH, whilst the zoonotic potential of schistosome species, particularly their ability to form hybrids and infect multiple hosts, underscores the need for a One Health approach [37]. Yet, critical knowledge gaps remain, particularly in our understanding of how these burdens interact across sectors. This consequently slows progress towards the WHO elimination targets and raises questions about whether current control and elimination metrics capture the lived realities of individuals living in endemic areas.

Accurately measuring the true impact of schistosomiasis is essential for informing cost-effectiveness analyses and ensuring comparability across different health conditions.

## Measuring the burden of schistosomiasis

Measuring disease burdens is essential for setting health priorities and evaluating interventions. Two widely used metrics, **disability-adjusted life years (DALYs)** and **quality-adjusted life years (QALYs),** capture the impact of disease on both mortality and morbidity [38]; both measures have been used in cost-utility analyses of MDA programmes targeting human schistosomiasis [33]. These measures, along with HRQoL instruments, provide the foundation for economic evaluations and policy decisions (Table 1).

**Table 1:** Comparison of common HRQoL instruments: domains and preference-based properties

Instrument	Target age (years)	Dimensions covered
EQ-5D-3L/5L 39, 40	16 and older	Mobility, self-care, usual activities, pain/discomfort, anxiety/depression
EQ-5D-Y [41]	8–15	Mobility, self-care, usual activities, pain/discomfort, anxiety/depression
PedsQoL [42]	2–18 (different versions for infants, toddlers, children, and adolescents)	Physical functioning, physical symptoms, emotional functioning, social functioning, school/work functioning, and cognitive functioning (infant scales)
WHOQOL [43]	18 years and older	Physical health, psychological health, social relationships, and environment
SF-12 [44]	18 years and older	Physical health, psychological health, social relationships, and environment
SF-36 [45]	14 years and older	Physical functioning, role physical, role emotional, vitality, mental health, social functioning, bodily pain, and general health

EQ-5D-3L/5L: EuroQol 5 Dimensions 3-Level or 5-Level version; EQ-5D-Y: EuroQol 5 dimensions—youth version; SF-12: Medical Outcomes Study 12-Item Short Form Health Survey; SF-36: Medical Outcomes Study 36-Item Short Form Health Survey.

**Figure 2:**
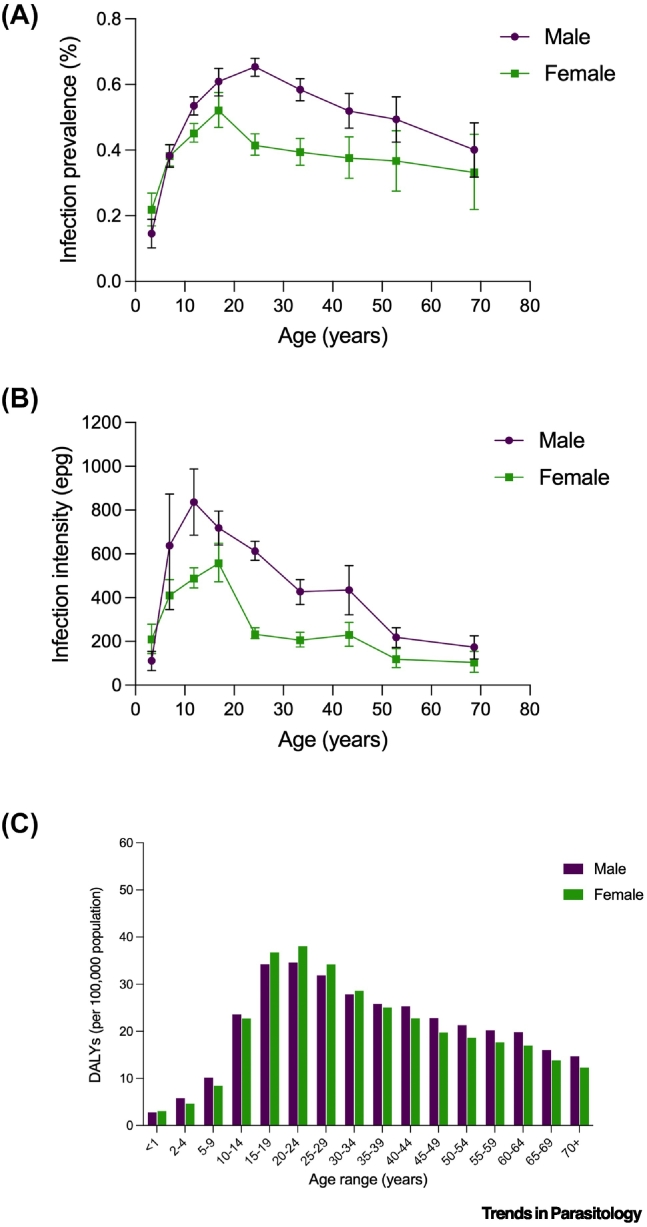
Schistosomiasis prevalence, intensity, and associated disability-adjusted life years (DALYs) over time. (A) Prevalence of *S. mansoni* (egg detection by microscopy) by age and sex. Bars represent exact binomial 95% CIs (data from a survey of approximately 10 000 individuals in Uganda [49]; data extracted using WebPlotDigitizer V5ii). (B) *S. mansoni* infection intensity by age and sex. Bars represent standard errors (data from a survey of approximately 10 000 individuals in Uganda [49]; data extracted using WebPlotDigitizer V5ii). (C) Global DALYs per 100 000 population attributable to schistosomiasis, stratified by age and sex (data from IHME 2021iii). DALYs: disability-adjusted life years; epg: eggs per gram; IHME: Institute for Health Metrics and Evaluation.

Global estimates suggest schistosomiasis caused over 12 800 deaths and 1.75 million DALYs in 2021, with nearly 88% of the burden in sub-Saharan Africa [46]. Despite ranking as one of the leading causes of disability among all NTDs in low-income countries [47], these figures still likely underestimate the true burden of schistosomiasis. For instance, DALY estimates rely on limited disability weights that do not adequately reflect common but debilitating disease manifestations, including genital schistosomiasis, chronic fatigue, pain, or anaemia [48]. Moreover, DALYs struggle to account for long infection-to-disease time lags and cumulative morbidity arising from repeated exposure, particularly when mortality is low but disability persists over decades 3, 24. As a result, schistosomiasis consistently appears less burdensome than conditions with more acute or fatal outcomes, despite its profound effects on wellbeing and productivity 6, 22, 48. These limitations are illustrated by the divergence between examples of *Schistosoma* age-specific prevalence, intensity, and DALYs lost, which highlight how cumulative morbidity persists beyond peak infection periods (Figure 2).

QALYs, whilst better suited for evaluating intervention benefits, face similar challenges. HRQoL instruments (often used to generate QALYs), including the EuroQol 5 Dimensions (EQ-5D) [50], World Health Organization Quality of Life (WHOQOL) [51] and Pediatric Quality of Life Inventory (PedsQoL) [52], have been used in schistosomiasis research but often exhibit strong ceiling effects in endemic settings. For example, in our recent study, 42% of participants reported ‘full health’ despite approximately two-thirds having parasitic infections and symptoms [50]. This likely reflects habituation to chronic ill-health, which may be due to both psychosocial adaptation to persistent symptoms and limitations in the sensitivity of generic HRQoL instruments, highlighting the need for careful interpretation of HRQoL estimates when informing policy and intervention thresholds [53]. Cultural differences in health perception and the limited sensitivity of generic instruments further underscore the need for refined measurement approaches to produce unbiased HRQoL/QALY estimates 50, 53. Furthermore, collecting and reporting societal outcomes, such as worsening school and productivity outcomes due to disease exposure, provide a wider picture of the overall impact of infection and can inform policy by complementing the measurement limitations of traditional health outcomes and HRQoL in these settings, as well as including any downstream effects despite potential psychosocial adaptation.

One potential approach to addressing this challenge is to assess biopsychosocial outcomes at both individual and community levels before and after treatment, which may help reveal otherwise unrecognised morbidity in endemic settings where chronic symptoms are normalised, and could be applied at both individual and community levels following MDA or other interventions.

Despite these limitations, such standard HRQoL instruments remain important for economic evaluations as they enable comparison of health outcomes across diseases and interventions. One potential approach is to complement generic instruments with disease-specific modules that are more sensitive to schistosomiasis-related morbidity, whilst preserving cross-disease comparability for decision-making regarding competing health priorities. These could be further enhanced by context-adapted measures, such as expanding HRQoL or other non-disease-specific tools to include questions that are locally relevant for areas commonly endemic to diseases of poverty, such as schistosomiasis, but not specifically focused on any individual disease or symptom. Informal mechanisms such as symposia or working groups could provide a pragmatic first step towards developing consensus protocols for core outcome measures across endemic settings.

Where morbidity is advanced, HRQoL decrements are clear. Morbidity from chronic *Schistosoma japonicum*, including hepatomegaly and hepatic fibrosis in China, was associated with reduced HRQoL (disability weights 0.19–0.447). Similar patterns have been observed for severe morbidity due to *Schistosoma mansoni* in Uganda (disability weights 0.20–0.44) for gastrointestinal bleeding and haematemesis [54]. However, studies examining the link between HRQoL and infection, rather than symptoms, and in particular mild schistosome infection in sub-Saharan Africa, are limited, and findings remain inconclusive. Whilst some report negative correlations in both adults and children 20, 55, 56, others have found no association 50, 57, 58. These inconsistencies may reflect differences in study design (i.e., community-based vs. health facility-based studies where individuals may present with different disease stages), age- or environmental-related HRQoL decline, instrument comprehension challenges, especially among children 42, 59 and instruments not being developed or optimised specifically for these populations. Ultimately, symptom severity seems to be a better predictor of HRQoL than infection status or intensity [50]; this has major policy implications, as current guidelines rely heavily on infection metrics.

Notably, at the time of this review, there was no data examining the impact of FGS/MGS on HRQoL, despite its recognised role in severe morbidity and complications [8].

If existing HRQoL measures, such as the EQ-5D and PedsQoL, are to be used for schistosomiasis, studies are needed to refine these tools so that they focus more on all aspects that may be affected by schistosomiasis, account for infection-to-disease time lags, and reflect socio-economic and epidemiological contexts.

## Critical gaps

### Rethinking the elimination of schistosomiasis as a public health problem

The WHO aims to achieve the elimination of schistosomiasis as a public health problem (EPHP) by 2030, defined as over 1% prevalence of heavy-intensity infections at the community level [60], with over 5% prevalence of heavy infection considered successful morbidity control [61]. Infection intensity categories based on parasitological egg counts in urine or faeces underpin these targets and serve as key indicators for national control programmes [62]: light [1–99 eggs/gram (epg) of stool for intestinal infections or <50 eggs/10 ml of urine for urogenital infections], moderate (100–399 epg), and heavy (≥400 epg or ≥ 50 eggs/10 ml of urine or visible haematuria) [1].

While this offers a practical framework for monitoring, the approach overlooks the complex, nonlinear relationship between infection and morbidity, time lags, and residual pathology from nonexcreted eggs. Recent analyses have also questioned whether heavy-intensity prevalence targets alone completely capture disease-related morbidity patterns in the era of large-scale control programmes, highlighting the need for complementary indicators that better reflect both infection prevalence and morbidity [63].

Diagnostic challenges further compound the problem. Conventional microscopy, used to categorise infection intensity, often misses low-intensity infections, particularly in post-MDA settings [64], leading to underestimation of prevalence and reduced MDA frequency, despite the potential persistence of morbidity from light infections. Evidence suggests that praziquantel reduces worm fecundity, but resurgence may be missed by egg-count–based follow-ups, enabling transmission and morbidity to continue [65]. One proposed solution is repeated egg microscopy measures or hatch tests using larger volumes [66]. Moreover, most schistosomiasis-related pathology is driven by nonexcreted eggs [67], with residual eggs trapped in intestinal or bladder tissue driving chronic inflammation and morbidity [7].

Therefore, in endemic areas where coinfections and comorbidities are common, understanding the fraction of morbidity attributable to schistosomiasis becomes imperative.

Improved diagnostic and monitoring tools are also recognised as a priority by the WHO to support schistosomiasis control and elimination programmes, particularly for detecting low-intensity infections and linking infection dynamics with morbidity outcomes.

Whilst research is still ongoing to better define the relationship between prevalence, infection intensity, and morbidity, multiple operationally validated indicators could be implemented at scale. For urogenital pathology, these include urine dipstick microhaematuria and proteinuria, and urine-albumin-to-creatinine ratio, whilst for intestinal or hepatic pathology, faecal occult blood and calprotectin provide point-of-care measures of mucosal bleeding and inflammation, and standardised WHO Niamey ultrasound grading enables consistent assessment of periportal fibrosis and other structural sequelae 68, 69, 70. The performance of these markers could be benchmarked against existing standards using criterion validity against ultrasound-defined pathology, treatment responsiveness, and latent-class analysis models that do not rely solely on egg-based indices.

Nonetheless, many available indicators remain species specific, resource intensive, and/or insufficiently sensitive to detect early or subtle morbidity. Advanced diagnostic tools, including point-of-care and molecular methods, are needed to quantify morbidity, measure treatment resistance, detect persistent infection, predict long-term outcomes, and link morbidity to standardised health metrics, including HRQoL and life expectancy, for better burden assessment.

Ultimately, focusing on heavy infections risks neglecting vulnerable groups such as preschool- and school-aged children, as well as maternal populations, the majority of whom carry light infections [71]. If we are to achieve EPHP by 2030 or beyond [61], there is a need to thoroughly review all of the targets delineated in the WHO NTD roadmap and adopt strategies that address all levels of infection intensity and actual morbidity, in order to address the remaining barriers to effective control and elimination. To reconsider the current EPHP thresholds, there is a need for extensive evidence that morbidity persists despite reaching heavy-intensity infection targets, and that alternative indicators better predict clinically and programmatically relevant outcomes. Such measures should complement, not replace, simple programme indicators to preserve accountability and comparability.

### EPHP and HRQoL

Although infection intensity is used as a proxy for disease burden, accumulating evidence suggests that morbidity is a stronger predictor of reduced HRQoL than egg counts alone 50, 63, creating a disconnect between current programmatic targets and the lived experiences of affected populations. For instance, whilst high-intensity *S. haematobium* infections consistently show a clear association with morbidity prevalence, their effect on HRQoL is inconsistent 11, 52, 57. Conversely, *S. mansoni* infection intensity shows limited association with morbidity [72], yet even low-intensity infections have been associated with measurable changes in HRQoL values 50, 55 and disability [24].

These findings highlight critical gaps. First, current tools fail to capture the complex species- and age-specific morbidity associated with schistosomiasis, limiting the ability to predict morbidity and estimate accurate HRQoL values [63]. Additionally, infection intensity alone may overlook symptoms such as fatigue, pain, diarrhoea, and anaemia, arising from other pathological processes such as granuloma formation, mucosal damage, and chronic inflammation; these may persist despite low egg counts and cumulatively reduce HRQoL [73]. As discussed previously, schistosomiasis can contribute to long-term disability and productivity losses, both key factors that collectively reinforce cycles of poverty and reduced quality of life. How best to capture these broader impacts remains unresolved.

### Impact of coinfections and comorbidities on HRQoL

Coinfections with soil-transmitted helminths (STHs), malaria, and HIV are common in schistosomiasis-endemic settings, yet the mechanistic processes of how coinfections exacerbate the health outcomes of schistosomiasis are poorly understood [74]. For instance, HIV coinfection can impair immune-mediated schistosome egg excretion, potentially increasing tissue pathology [75] whilst simultaneously underestimating infection intensity. Additionally, praziquantel efficacy may also be reduced, with treatment primarily affecting fecundity whilst leaving schistosomes metabolically active [76]. In contrast, the relationship with STH appears more variable, with studies reporting both improved and worsened outcomes 77, 78; another indication of the need to understand the immunopathology of coinfections with schistosomiasis.

Despite growing interest, there is uncertainty regarding how coinfections shape disease progression, treatment outcomes, and HRQoL [74]. Disentangling morbidity attributable to schistosomiasis in endemic settings can be challenging, primarily because common coinfections and shared structural determinants such as poverty, nutrition, and environmental exposure may produce overlapping symptoms and health outcomes. Epidemiological approaches such as estimating population or morbidity attributable fractions, commonly used in burden of disease analyses, may help quantify the proportion of morbidity directly associated with schistosomiasis [79]. Even more challenging is the fact that determining morbidity attributable fractions does not indicate causation *per se*. Thus, applying such methods requires robust longitudinal datasets, integrated diagnostics for multiple infections, and accounting for multiple socio-economic and environmental confounders. Strengthening such evidence will be essential for designing integrated, context-appropriate, and horizontal disease network approaches to address overlapping disease burdens, improve causal attribution, and gain accurate estimates of the true burden of schistosomiasis. By integrating evidence from diverse studies and settings, meta-analysis can provide more precise estimates of the relationship between light infection and morbidity that can inform epidemiological models and improve monitoring and evaluation [80].

### Socio-economic aspects

Children, individuals living near water bodies, and those engaged in farming or fishing are at the highest risk of schistosomiasis due to frequent water contact [2]. However, the long-term impact of schistosomiasis on HRQoL appears greater among adults, particularly in chronic disease [50]. Nevertheless, the role of age, gender, religion, education, and socio-economic status in shaping infection risk remains inconclusive, as water contact often reflects cultural practices and gender norms, as well as education level or wealth 2, 81. Additionally, evidence suggests that higher socio-economic status is correlated with reduced exposure and schistosomiasis prevalence [82]. Yet, in rural sub-Saharan Africa, where household socio-economic conditions may be relatively similar, the extent to which poverty drives infection and disease remains unclear [83]. These uncertainties complicate efforts to design interventions that address both transmission and its structural determinants.

### Psychological aspects

Research explicitly examining mental health outcomes associated with schistosomiasis remains sparse. A major challenge is the lack of standardised psychological outcome measures for schistosomiasis, which limits comparability and hinders efforts to build a more robust shared evidence base for understanding and addressing mental health problems [84]. A small number of studies have employed validated instruments such as the Patient Health Questionnaire (PHQ-9) [85]. There is a need for researchers to consolidate and agree on appropriate psychological measures, ideally codesigned with communities to ensure lived-experience, cultural relevance, and sensitivity to disease-specific and broader mental health impacts.

Although praziquantel is safe and effective, MDA alone may not be enough to address the psychological sequelae of schistosomiasis. There is a need for integrated approaches that combine medical, mental health, and social interventions, particularly for chronic disease and across different age groups.

### Economic evaluation of treatment/preventive chemotherapy

Economic evaluations of schistosomiasis remain scarce, often limited in scope and marked by important methodological shortcomings 33, 86. Whilst MDA has been deemed ‘cost-effective’ in some cases [87], its impact on morbidity and HRQoL remains unclear [88]. To date, no study has adopted a lifetime horizon to capture cumulative morbidity from repeated exposure, despite links to chronic complications and the fact that current infection rarely equates to cumulative morbidity. Moreover, most analyses rely on DALYs and rarely adopt a societal perspective, thereby limiting their relevance for equity-oriented policy design [86].

Further challenges include the lack of generic and/or disease-specific health outcomes to evaluate interventions or justify WASH investments. Inconsistent HRQoL findings hinder the use of standard metrics in economic models, and the lack of epidemiological models linking short- and long-term infection with morbidity limits accurate assessment. Robust long-term cohort and modelling studies are needed to assess both morbidity and societal impacts of schistosomiasis, including productivity and educational outcomes. partnerships such as the SchistoTrack Cohort, established in 2022, are beginning to address some of these knowledge gaps through a longitudinal, community-based study of over 4500 participants in Uganda [89]. Uncertainties remain regarding how best to capture lifetime and societal impacts.

### Spillover effects of infection and morbidity

To the best of our knowledge, there is no evidence on spillover effects of *Schistosoma* infection at household or community levels. This gap is critical because morbidity in infected family members likely generates indirect costs through lost productivity, caregiving burden, and psychological stress. We hypothesise that these spillover effects extend beyond economics to psychological health and HRQoL; families may experience anxiety about illness, income loss, or their own risk of infection. This may be particularly relevant in schistosome-endemic settings with limited healthcare access, frequent reinfection, and minimal social protection. Vulnerable individuals reliant on subsistence farming may be unable to work, bring food home (increasing food insecurity risks), or access proper and timely treatment; these realities make a strong case for implementing universal health coverage systems [90].

Understanding the magnitude and reach of these indirect effects is essential for estimating the true burden of schistosomiasis and the potential gains from elimination. Establishing causal spillover effects requires approaches that identify health and economic changes at household and community levels caused by infection or treatment of others within those households and/or communities. Study designs, including longitudinal cohort studies, randomised trials, and natural or quasi-experimental approaches (e.g., instrumental variables, regression discontinuity, or difference-in-differences), are ideally suited for this purpose, especially when they integrate epidemiological, economic, and psychosocial data across households and communities.

### Key questions

Taken together, these gaps highlight fundamental questions regarding how schistosomiasis is currently measured, prioritised, and addressed, particularly in relation to morbidity, recovery, HRQoL, and the broader social and economic impacts.

Our ongoing study, WickedSchisto,i established in 2024, will address some of these gaps through a large-scale interdisciplinary initiative in Uganda and Cameroon, combining WASH infrastructure, access and use, longitudinal *S. mansoni* epidemiological, socio-economic, capability wellbeing, HRQoL, and mental health assessments with qualitative findings on what a good quality of life is. This will help quantify the impact of *S. mansoni* on individuals, households, and communities, including spillover in a locally relevant manner, and inform cost-effectiveness analyses of WASH- and MDA-based interventions.

## Pitfalls and methodological challenges in current research

The WHO NTD Strategic and Technical Advisory Group recognises that ‘research and innovation are fundamental enablers of programmatic progress for all NTDs’ [61]. However, the current schistosomiasis research landscape is marked by a myriad of challenges that limit accurate burden estimation, comparison across settings, and evaluation of interventions (Table 2). Central among these is the delayed and nonlinear relationship between infection, morbidity, and impact on HRQoL [3], which undermines short-term assessments and egg-based definitions of cure. In fact, decades of work in both human and experimental models have provided valuable insights into disease aetiology, yet these paradigms are difficult to extrapolate to human studies, partly due to differences in infection dynamics across age groups and endemicity levels [3]. These limitations are compounded by short study horizons, insufficient attention to recovery trajectories, and the underuse of community-informed approaches. Together, these issues contribute to the systematic underestimation of chronic, psychosocial, and socio-economic impacts.

**Table 2:** Key pitfalls and methodological challenges in schistosomiasis research

Pitfall/challenge	What goes wrong	Why it matters	What is needed
Early infection and asymptomatic stages	Early or low-intensity infections (especially in low-prevalence settings) are frequently missed by conventional diagnostics	Missed diagnoses delay treatment and enable progression to chronic morbidity	Develop and adopt more sensitive and specific diagnostics (e.g., CAA/CCA, molecular tools); use multitest strategies tools (aligned with WHO target product profiles) that detect early and light infections and predict long-term outcomes
Delayed response between infection and morbidity	Morbidity often manifests months to years after infection, whilst diagnostics focus on current egg excretion	Complicates our understanding and measurement of the true burden/impact of disease, as well as the assessment of intervention effectiveness	Shift focus towards markers of delayed and cumulative exposure/morbidity, residual pathology, and future morbidity risk rather than relying on current infection status alone; addressing these temporal dynamics will likely require longitudinal cohort data combined with modelling approaches that link cumulative infection exposure with delayed morbidity and recovery trajectories, whilst using simple delay terms and spline-based functions to capture nonlinear progression without making the model too complex
Nonspecific morbidity markers	Classic indicators (e.g., haematuria, diarrhoea, and anaemia) lack specificity, causal attribution, and the ability to predict long-term morbidity	Difficult to disentangle schistosomiasis-related morbidity from coinfections or comorbidities; limits insight into subtle or long-term effects of infection on all aspects of overall health	Combine diagnostics with novel biomarkers from molecular diagnostics, imaging techniques, and bioinformatic; long-term follow-up to improve attribution; expand morbidity metrics beyond infection (e.g., to include mental health, nutrition, cognition, socio-economic status, and HRQoL)
Limited prognostic value of diagnostics	EPHP is assessed using infection as a proxy for morbidity thresholds only; many tests indicate infection presence but not disease trajectory	Morbidity is not fully correlated with intensity; inability to identify individuals at highest risk of long-term disability	Develop and prioritise diagnostics with prognostic value for chronic pathology and recovery
Short-study time horizons	Most studies capture outcomes over weeks/months rather than years	Failure to capture long-term impact of infection/disease and benefits of treatment or interventions; failure to understand the trajectory of recovery/morbidity reversal posttreatment	Invest in long-term longitudinal cohorts tracking infection, morbidity resolution, and reinfection dynamics; integrate clinical, socio-economic, and wellbeing outcomes into studies
Resource and logistical constraints	Long-term follow-up studies can be costly, complex, and difficult to sustain; accompanied by ethical and logistical complexities (e.g., cultural/socio-political barriers, lack of infrastructure and health resources or personnel	Bias towards short-term, studies and biomedical outcomes	Sustained and longer-term funding to support innovative and flexible study designs as well as studies that can leverage advancements in diagnostic, data collection, and analytical techniques
Limited community involvement	Research questions and metrics may not reflect lived experiences	Cultural misalignment of research tools and interventions; reduced relevance and uptake of interventions; challenges in trust building, sustainability, and balancing scientific rigour with community priorities	Embed community-based participatory approaches in study design and evaluation; incorporate qualitative research to better understand lived experiences of affected individuals, and ensuring findings are meaningfully disseminated back to communities and stakeholders, and can help strengthen local engagement and policy impact
Data collection and reporting bias	Selection bias (excluding hard-to-reach populations), reporting bias (due to stigma or cultural norms), and measurement bias (from diagnostic limitations) may skew prevalence and morbidity estimates	Inequitable policies and misallocation of resources	Improve sampling strategies to include marginalised populations; adopt culturally sensitive data collection methods; standardise and validate data collection tools; be transparent in reporting uncertainty and bias
Incomplete assessment of recovery	Curative treatment is often defined by the absence of parasite eggs in stool/urine and less morbidity resolution	Persistent morbidity or disability and reduced HRQoL may be overlooked post-treatment	Redefine treatment success to include functional recovery and improvement in overall HRQoL
Inadequate disease burden metrics	DALYs and QALYs may fail to capture mild, chronic, psychosocial, and social impacts of disease	Underestimation of true disease burden and the value of interventions, especially at the individual level	Develop and implement multidimensional health indices that include physical, psychological, and social health indicators
Disconnect between health outcomes and costs	The impact of disease on health versus economic consequences is often analysed separately	Weak economic evaluations and limited comparability across interventions	Integrate health, economic, and social outcomes within unified evaluation frameworks
What constitutes a ‘public health problem’?	No clarity on what constitutes ‘locally acceptable’ morbidity levels for schistosomiasis to be actually eliminated as a public health problem	Ambiguity in monitoring EPHP success; risks overlooking persistent physical, psychological, or social morbidity despite meeting infection-based targets; weakens relevance of EPHP benchmarks for affected communities and policymakers	Use qualitative and mixed-methods research to define context-specific morbidity thresholds; integrate lived experience, functional outcomes, and wellbeing into EPHP frameworks; operationalise such insights into measurable indicators that complement current infection metrics

CAA: circulating anodic antigen; CCA: circulating cathodic antigen.

To capture the true impact of schistosomiasis, research must account for the complexities introduced by the pathophysiology of the disease, as well as its ramifications on people’s perceptions of the disease, morbidity, and HRQoL.

## Conceptual framework for advancing schistosomiasis research and control

**Figure 3:**
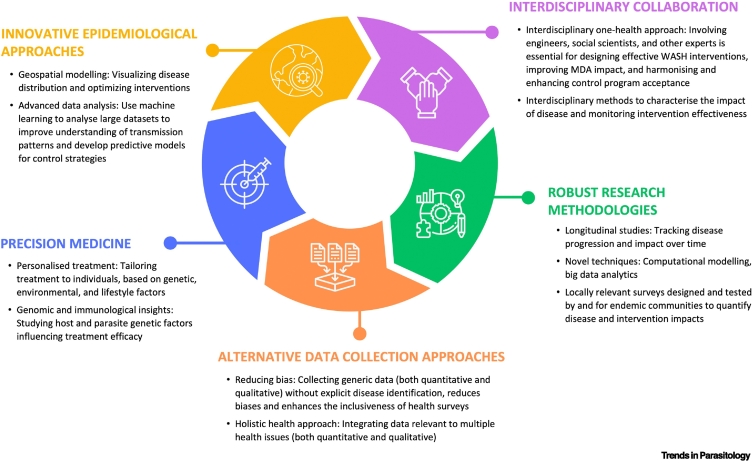
Key figure. Conceptual framework for advancing schistosomiasis research and control. MDA: mass drug administration; WASH: water, sanitation, and hygiene.

Taken together, we propose a conceptual framework that integrates methodological advances across health, behavioural, and biological domains to better capture, understand, and respond to the complex impact of schistosomiasis (Figure 3, Key figure). This framework illustrates how linking these methods can help identify gaps, improve measurements, and inform equitable and holistic intervention strategies aligned with elimination and development goals. Such a framework is not intended to replace the key indicators required for routine programme delivery but rather to complement them by strengthening research, evaluation, and long-term policy design, which aligns with current guidelines on multicriteria decision analysis that prioritises interventions based on different criteria [91].

Advancing schistosomiasis research requires robust, interdisciplinary approaches that integrate biological, social, and environmental dimensions. An interdisciplinary One Health approach has been proposed to conduct research and to design and develop national programs [92]; this should be viewed as a complementary rather than competing approach, enabling different disciplinary perspectives to jointly inform a more comprehensive understanding of disease impacts [91]. For example, engineers and social scientists have worked together to design new WASH interventions tailored to local communities, demonstrating how multisectoral engagement improves program acceptance and effectiveness [93]. However, multisectoral and multidisciplinary collaboration requires overcoming challenges related to establishing clear communication on the benefits of collaboration for each sector, aligning terminologies, institutionalising partnerships within policy frameworks, and identifying cross-cutting progress indicators to track progress and scale public health impact [93].

As noted earlier, biases in data collection and reporting can compromise burden estimates, intervention design, and public health decision-making. Disease-specific framing of data collection tools may often shape researcher priorities and influence participant responses and behaviours; for example, researchers may prioritise disease-specific symptoms, and participants may over-report water contact due to recall bias, alter behaviours (e.g., reducing water contact), or under-report by providing responses they think the researcher wants to hear [94]. Alongside vitally important open and nonjudgemental interviewing, collecting more generic data through neutral questions (e.g., daily activities involving water) may help reduce recall and interviewer bias by masking the study’s intent [95]. Such approaches can help gather information on general health, economic status, and social conditions (e.g., general morbidity, absenteeism, and economic productivity), which can later be correlated with epidemiological, biological, or mechanistic data on schistosomiasis. Moreover, generic tools support a horizontal research model that addresses multiple health issues simultaneously, improving cost-effectiveness and fostering community engagement by focusing on overall wellbeing rather than on a single disease. In the case of schistosomiasis, endemic communities are more likely to actively participate in health programs that consider their overall wellbeing rather than focusing on a single disease [96].

Advancements in genomics, proteomics, immunogenetics, and personalised medicine offer new avenues for schistosomiasis control by accounting for host and parasite variability that influences the risk of infection and treatment response. In the context of schistosomiasis, this will involve understanding the parasite and host genetic, pharmacological, and immunological factors that influence the efficacy of treatments such as praziquantel. For example, variability in liver metabolism, drug–drug interactions, and genetic variations in cytochrome P450 enzymes can impact praziquantel metabolism, affecting cure rates and treatment success [97]. Additionally, immunogenetic studies reveal associations between specific alleles and infection risk. For example, the *IL4 rs2070874T* allele has been linked with protection against schistosomiasis, and the *IFNG rs2069727G* allele with increased susceptibility [98]. Integrating these insights into treatment strategies could improve cure rates, move schistosomiasis care a step closer towards precision medicine, and enhance intervention effectiveness. Our new project, DRIVERS, aims to address some of these individual-level drivers of treatment failures to inform recommendations to improve drug efficacy.

Finally, innovative epidemiological approaches such as geospatial modelling can help in visualising the distribution of schistosomiasis, understanding transmission dynamics, and optimising resource allocation for interventions. For example, integrating remote sensing data with geographic information systems can identify water bodies that are potential snail habitats, predict outbreak zones, hotspot areas, and guide targeted interventions [99]. Additionally, modelling and machine learning algorithms can process large datasets to identify patterns and predictors of transmission that might not be apparent through traditional methods, leading to the design of more effective control strategies [100]. These two approaches will be incorporated into DRIVERS, helping to inform on the community-level drivers of persistent transmission.

## Concluding remarks and future perspectives

Current infection intensity does not equate to current morbidity. We need new markers to detect early stages of morbidity and morbidity in low-intensity infections, especially for *S. mansoni* and genital schistosomiasis. We need to understand what constitutes a public health problem by considering all dimensions affecting the health and lives of individuals in endemic communities, and how best we can measure them to fully understand the wider impact of schistosomiasis and, therefore, monitor progress towards the WHO elimination targets. Expanding the definition of true impact should be anchored to clear morbidity metrics and transparent reporting frameworks, such that the broader health and economic benefits can be highlighted without diluting disease-specific accountability or weakening elimination efforts.

To do this, further research is needed to address the knowledge gaps regarding the effect of low, moderate, and heavy schistosome infections on mental health, capability wellbeing, and overall quality of life (see Outstanding questions). These outcomes must be measured using instruments relevant to those living in endemic communities, such as WickedSchisto, which collects data on mental health, capability wellbeing, HRQoL economic costs, and spillover, alongside extensive data on infection, coinfection, and the impact of WASH access and use on these measures. Detailed longitudinal cohort studies are needed to ensure that we can accurately understand the long-term horizons for the development of morbidity markers that are transferable across health issues, as well as downstream economic costs such as school attainment and productivity. For example, SchistoTrack, which collects prospective data to better understand the transmission and morbidity of schistosomiasis [89]. These and new studies will help fill the existing evidence gap in understanding the true impact of the disease whilst informing economic evaluations of different interventions across competing health issues, and also taking spillovers into account to better understand the wider and long-term implications of individual, community, and national infection presence. This will facilitate a better understanding of the public health problem and likely strengthen arguments for investing large upfront costs in key interventions (such as WASH infrastructure and maintenance) that will sustain reductions in disease burden and increase economic gains at the individual, community, and national levels over time, helping to raise populations out of poverty (see **Outstanding questions**).

Outstanding questions What is the ‘true’ burden of schistosomiasis?

How should the elimination of schistosomiasis as a public health problem be defined and measured beyond heavy-intensity infection thresholds? How can these targets better reflect morbidity, recovery, and long-term health outcomes?

Why do some individuals develop morbidity with light-intensity infections, whilst others remain apparently asymptomatic despite high infection intensity? How do time lags and residual pathology affect these outcomes?

How can light-intensity and chronic infections be reliably detected and linked to meaningful health outcomes?

How can we measure the multifaceted impacts of schistosomiasis to guide resource allocation and cost-effective interventions across competing health issues? How can morbidity be more accurately captured and linked to standardised measures of wellbeing and health-related quality of life? Could complementary approaches, such as capability or psychometric instruments, improve measurement and comparability across settings?

How can health-related quality of life be better integrated into routine monitoring and evaluation of control programmes?

What roles do coinfections, comorbidities, and social contexts play in shaping disease progression, treatment response, and health-related quality of life?

Is preventive chemotherapy alone sufficient to address the psychological and social consequences of schistosomiasis? What integrated care models are feasible in resource-limited settings?

How do socio-economic conditions and livelihoods shape long-term health and productivity losses associated with schistosomiasis?

How can economic evaluations better incorporate lifetime horizons, societal perspectives, and household- and community-level spillover effects?

How can models link infection trajectories with long-term morbidity to inform policy and investment decisions?

What combinations of preventive chemotherapy; water, sanitation, and hygiene; and health system interventions deliver the greatest long-term benefits?

Alt-text: Outstanding questions

## Glossary

**Disability-adjusted life years (DALYs)**: a measure of disease burden combining years of life lost due to premature death and years lived with disability, weighted by disease severity. DALYs are commonly used to quantify the overall health loss in a population and are widely utilised to compare disease burden across conditions.
**Female and male genital schistosomiasis (FGS/MGS)**: chronic complications of schistosomiasis characterised by the presence of trapped parasite eggs (primarily by *S. haematobium* and rarely by *S. mansoni*), resulting in pathologies in the reproductive fluids (males) and genital organs.
**Health-related quality of life (HRQoL)**: a multidimensional concept that reflects how ill-health affects an individual’s perceived sense of physical, mental, and social wellbeing. HRQoL measures an individual’s perception of their physical and mental health over time.
**Mass drug administration (MDA)**: a form of preventive chemotherapy and a public health strategy in which populations within a specific geographical area (typically endemic for a disease) are given medical treatment for the disease all at once, regardless of whether individuals show symptoms or have a confirmed diagnosis.
**Neglected tropical diseases (NTDs)**: a diverse group of approximately 21 conditions caused by various pathogens (this includes viruses, bacteria, parasites, and fungi). They primarily affect impoverished communities in tropical and subtropical regions.
**Quality-adjusted life years (QALYs)**: a measure of health outcomes that assigns utility values (e.g., 0 =death, 1 =full health) to time spent in different health states. QALYs reflect both the quantity and quality of life and are considered the gold standard for evaluating the benefits of health interventions, often derived from HRQoL data.
**Schistosomiasis**: a parasitic disease caused by trematode worms of the genus *Schistosoma. S. haematobium* primarily causes urogenital disease, whilst *S. mansoni* and *S. japonicum* cause intestinal disease. Transmission occurs when eggs excreted in urine or faeces reach freshwater, hatch into miracidia, and infect intermediate snail hosts, which then release cercariae that penetrate human skin. Diagnosis typically involves detecting parasite eggs in urine or stool or identifying circulating antigens in urine or serum. Infections can be treated with the anthelmintic drug praziquantel, though it does not prevent reinfection or reverse severe morbidity.

## Resources

^i^ https://www.openaccessgovernment.org/article/wicked-schisto-public-health-problem-schistosomiasis-interdisciplinary-research-control/150145/

^ii^ https://automeris.io/

^iii^ https://www.healthdata.org

## References

[1] World Health Organization (2022).

[2] World Health Organization (2026).

[3] McManus D.P. (2018). Schistosomiasis. Nat. Rev. Dis. Primers.

[4] Koopman J.P.R. (2021). Controlled human infection models to evaluate schistosomiasis and hookworm vaccines: where are we now?. Expert Rev. Vaccines.

[5] Bolton D. (2023). A revitalized biopsychosocial model: core theory, research paradigms, and clinical implications. Psychol. Med..

[6] Conteh L. (2010). Socioeconomic aspects of neglected tropical diseases. Lancet.

[7] Buonfrate D. (2025). Human schistosomiasis. Lancet.

[8] Bustinduy A.L., Rollinson D., Stothard R. (2022). Advances in Parasitology.

[9] Bustinduy A.L. (2013). Fecal occult blood and fecal calprotectin as point-of-care markers of intestinal morbidity in Ugandan children with *Schistosoma mansoni* infection. PLoS Negl. Trop. Dis..

[10] Wami W.M. (2015). Identifying and evaluating field indicators of urogenital schistosomiasis-related morbidity in preschool-aged children. PLoS Negl. Trop. Dis..

[11] Wiegand R.E. (2021). Associations between infection intensity categories and morbidity prevalence in school-age children are much stronger for *Schistosoma haematobium* than for *S. mansoni*. PLoS Negl. Trop. Dis..

[12] Adjorlolo S. (2024). Prevalence, assessment and correlates of mental health problems in neglected tropical diseases: a systematic review. Int. Health.

[13] Utzinger J. (2015). New diagnostic tools in schistosomiasis. Clin. Microbiol. Infect..

[14] Wade D.T., Halligan P.W. (2017). The biopsychosocial model of illness: a model whose time has come. Clin. Rehabil..

[15] Qi Y.-X. (2024). Prevalence of depressive symptoms in patients with advanced schistosomiasis in China: a systematic review and meta-analysis. PLoS Negl. Trop. Dis..

[16] Masong M.C. (2024). Illness experiences and mental health challenges associated with female genital schistosomiasis in Cameroon: a gender analysis. Int. Health.

[17] Mushi V., Tarimo D. (2022). Urogenital schistosomiasis knowledge, attitudes and practices among the community members in Lindi, Tanzania: a qualitative study. East Afr. J. Sci..

[18] Ahlberg B.M. (2003). ‘Better infection than hunger.’ A study of illness perceptions with special focus on urinary schistosomiasis in Northern Tanzania. Afr. Sociol. Rev..

[19] Ezeamama A.E. (2018). Cognitive deficits and educational loss in children with schistosome infection—A systematic review and meta-analysis. PLoS Negl. Trop. Dis..

[20] Kinung’hi S. (2016). Infection with *Schistosoma mansoni* has an effect on quality of life, but not on physical fitness in schoolchildren in Mwanza region, North-Western Tanzania: a cross-sectional study. PLoS Negl. Trop. Dis..

[21] Musuva R. (2017). Change in children’s school behavior after mass administration of praziquantel for *Schistosoma mansoni* infection in endemic areas of western Kenya: a pilot study using the behavioral assessment system for children (BASC-2). PLoS One.

[22] Lenk E.J. (2016). Productivity loss related to neglected tropical diseases eligible for preventive chemotherapy: a systematic literature review. PLoS Negl. Trop. Dis..

[23] Adeyemo P. (2022). Estimating the financial impact of livestock schistosomiasis on traditional subsistence and transhumance farmers keeping cattle, sheep and goats in northern Senegal. Parasit. Vectors.

[24] King C.H. (2005). Reassessment of the cost of chronic helmintic infection: a meta-analysis of disability-related outcomes in endemic schistosomiasis. Lancet.

[25] King C.H. (2015). It’s time to dispel the myth of “asymptomatic” schistosomiasis. PLoS Negl. Trop. Dis..

[26] Wilson Z. (2016). Out of the shadows-making mental health a global development priority. Mental Health Matters.

[27] Lampard-Scotford A.R. (2022). Impact of parasitic infection on mental health and illness in humans in Africa: a systematic review. Parasitology.

[28] Hofstraat K., van Brakel W.H. (2016). Social stigma towards neglected tropical diseases: a systematic review. Int. Health.

[29] Chen M.L. (2015). Qualitative research on psychological experiences of advanced schistosomiasis patients. Zhongguo Xue Xi Chong Bing Fang Zhi Za Zhi.

[30] Yong-Xin N., Tian-Liang X. (2012). Cognitive behavioral therapy for depression in advanced schistosomiasis patients. Chin. J. Schistosomiasis Control.

[31] Hua Haiyong H.H. (2010). Evaluation of quality of life in advanced schistosomiasis patients in Jiangsu province. Chin. J. Schistosomiasis Control.

[32] World Health Organization (2009).

[33] Turner H.C. (2020). Economic evaluations of human schistosomiasis interventions: a systematic review and identification of associated research needs. Wellcome Open Res..

[34] Salari P. (2020). Cost of interventions to control schistosomiasis: a systematic review of the literature. PLoS Negl. Trop. Dis..

[35] Hamory J. (2021). Twenty-year economic impacts of deworming. Proc. Natl. Acad. Sci. U. S. A..

[36] Bloom D.E. (2019). Health and economic growth. Oxford Res. Encyclopedia Eco. Fin..

[37] Borlase A. (2021). Spillover, hybridization, and persistence in schistosome transmission dynamics at the human-animal interface. Proc. Natl. Acad. Sci. U. S. A..

[38] Hyder A.A. (2012). Measuring the health of populations: explaining composite indicators. J. Public Health Res..

[39] Wisloff T. (2014). Estimating QALY gains in applied studies: a review of cost-utility analyses published in 2010. Pharmacoeconomics.

[40] Kennedy-Martin M. (2020). Which multi-attribute utility instruments are recommended for use in cost-utility analysis? A review of national health technology assessment (HTA) guidelines. Eur. J. Health Econ..

[41] Wille N. (2010). Development of the EQ-5D-Y: a child-friendly version of the EQ-5D. Qual. Life Res..

[42] Varni J.W. (2001). PedsQLPedsQL 4.0: reliability and validity of the Pediatric Quality of Life Inventory version 4.0 generic core scales in healthy and patient populations. Med. Care.

[43] World Health Organization (2012).

[44] Ware J.E. (1996). A 12-item short-form health survey: construction of scales and preliminary tests of reliability and validity. Med. Care.

[45] Ware J.E. (2008).

[46] Li Q. (2025). Global trends of schistosomiasis burden from 1990 to 2021 across 204 countries and territories: findings from GBD 2021 study. Acta Trop..

[47] GBD Disease Injury Incidence and Prevalence Collaborators (2017). Global, regional, and national incidence, prevalence, and years lived with disability for 328 diseases and injuries for 195 countries, 1990–2016: a systematic analysis for the global burden of disease study 2016. Lancet.

[48] King C.H., Galvani A.P. (2018). Underestimation of the global burden of schistosomiasis. Lancet.

[49] Kabatereine N.B. (2004). Epidemiology and geography of *Schistosoma mansoni* in Uganda: implications for planning control. Trop. Med. Int. Health.

[50] Alonso S. (2024). The short-term impact of *Schistosoma mansoni* infection on health-related quality of life: implications for current elimination policies. Proc. Biol. Sci..

[51] Roriz S.J. (2021). Quality of life assessment among patients living with hepatosplenic schistosomiasis and schistosomal myeloradiculopathy. Front. Med. (Lausanne).

[52] Terer C.C. (2013). Evaluation of the health-related quality of life of children in *Schistosoma haematobium*-endemic communities in Kenya: a cross-sectional study. PLoS Negl. Trop. Dis..

[53] Eisenstein E.M., Eisenstein D. (2006). A behavioral homeostasis theory of habituation and sensitization: II. Further developments and predictions. Rev. Neurosci..

[54] Opio C.K. (2021). Hepatic schistosomiasis, upper gastrointestinal bleeding, and health related quality of life measurements from the Albert Nile Basin. J. Patient Rep. Outcomes.

[55] Furst T. (2012). Schistosomiasis, soil-transmitted helminthiasis, and sociodemographic factors influence quality of life of adults in Cote d’Ivoire. PLoS Negl. Trop. Dis..

[56] Olsen A. (2020). Changes in morbidity, physical fitness, and perceived quality of life among schoolchildren following four years of different mass drug administration strategies against *Schistosoma mansoni* infection in Mwanza region, Northwestern Tanzania. Am. J. Trop. Med. Hyg..

[57] Hürlimann E. (2014). Health-related quality of life among school children with parasitic infections: findings from a national cross-sectional survey in Côte d’Ivoire. PLoS Negl. Trop. Dis..

[58] Won K.Y. (2014). Assessment of quality of life as a tool for measuring morbidity due to *Schistosoma mansoni* infection and the impact of treatment. Am. J. Trop. Med. Hyg..

[59] Wang J. (2022). Health-related quality of life in children: the roles of age, gender and interpersonal trust. Int. J. Environ. Res. Public Health.

[60] World Health Organization (2020).

[61] World Health Organization (2022).

[62] World Health Organization (2013).

[63] Wiegand R.E. (2022). Defining elimination as a public health problem for schistosomiasis control programmes: beyond prevalence of heavy-intensity infections. Lancet Glob. Health.

[64] Doehring E. (1983). Day-to-day variation and circadian rhythm of egg excretion in urinary schistosomiasis in the Sudan. Ann. Trop. Med. Parasitol..

[65] Lamberton P.H.L. (2017). Praziquantel decreases fecundity in *Schistosoma mansoni* adult worms that survive treatment: evidence from a laboratory life-history trade-offs selection study. Infect. Dis. Poverty.

[66] Colley D.G. (2017). Schistosomiasis is more prevalent than previously thought: what does it mean for public health goals, policies, strategies, guidelines and intervention programs?. Infect. Dis. Poverty.

[67] Colley D.G. (2014). Human schistosomiasis. Lancet.

[68] Vaillant M.T. (2024). Diagnostic tests for human *Schistosoma mansoni* and *Schistosoma haematobium* infection: a systematic review and meta-analysis. Lancet Microbe.

[69] Shehab A.Y. (2023). Proposed morbidity markers among *Schistosoma mansoni* patients. Trop. Parasitol..

[70] Ockenden E.S. (2024). The role of point-of-care ultrasound in the assessment of schistosomiasis-induced liver fibrosis: a systematic scoping review. PLoS Negl. Trop. Dis..

[71] Bustinduy A.L. (2017). Paediatric and maternal schistosomiasis: shifting the paradigms. Br. Med. Bull..

[72] Wiegand R.E. (2021). Control and elimination of schistosomiasis as a public health problem: thresholds fail to differentiate schistosomiasis morbidity prevalence in children. Open Forum Infect. Dis..

[73] Murray C.J.L. (1996).

[74] Perera D.J. (2024). Beyond schistosomiasis: unraveling co-infections and altered immunity. Clin. Microbiol. Rev..

[75] Furch B.D. (2020). Interactions of *Schistosoma* and HIV in Sub-Saharan Africa: a systematic review. Am. J. Trop. Med. Hyg..

[76] Kallestrup P. (2006). Schistosomiasis and HIV in rural Zimbabwe: efficacy of treatment of schistosomiasis in individuals with HIV coinfection. Clin. Infect. Dis..

[77] Geiger S.M. (2008). Immuno-epidemiology of *Schistosoma mansoni* infections in endemic populations co-infected with soil-transmitted helminths: present knowledge, challenges, and the need for further studies. Acta Trop..

[78] Abruzzi A., Fried B. (2011). Coinfection of *Schistosoma* (Trematoda) with bacteria, protozoa and helminths. Adv. Parasitol..

[79] Fernandez K. (2025). Population attributable fraction: translating causal knowledge into prevention potential. Eur. J. Pub. Health.

[80] Ewuzie A. (2025). Association of current *Schistosoma mansoni*, *Schistosoma japonicum*, and *Schistosoma mekongi* infection status and intensity with periportal fibrosis: a systematic review and meta-analysis. Lancet Glob. Health.

[81] Ayabina D.V. (2021). Gender-related differences in prevalence, intensity and associated risk factors of *Schistosoma* infections in Africa: a systematic review and meta-analysis. PLoS Negl. Trop. Dis..

[82] Muhumuza S. (2009). Association between socio economic status and schistosomiasis infection in Jinja district, Uganda. Trop. Med. Int. Health.

[83] Omondi I. (2021). Socioeconomic determinants of *Schistosoma mansoni* infection using multiple correspondence analysis among rural western Kenyan communities: evidence from a household-based study. PLoS One.

[84] Jacobs P. (2023). A scoping review of mental health and wellbeing outcome measures for children and young people: implications for children in out-of-home care. J. Child Adolesc. Trauma.

[85] Hu A. (2024). Depression in the schistosomiasis japonica population based on the PHQ-9 scale: a cross-sectional survey from Jiangxi Province, China. Sci. Rep..

[86] Uzoegbo S.C. (2022). A systematic review and quality appraisal of the economic evaluations of schistosomiasis interventions. PLoS Negl. Trop. Dis..

[87] Lo N.C. (2015). Comparison of community-wide, integrated mass drug administration strategies for schistosomiasis and soil-transmitted helminthiasis: a cost-effectiveness modelling study. Lancet Glob. Health.

[88] Abudho B.O. (2020). Evaluation of morbidity in *Schistosoma mansoni*-positive primary and secondary school children after four years of mass drug administration of praziquantel in western Kenya. Infect. Dis. Poverty.

[89] Big Data Institute (2025).

[90] Gruninger S. (2022). Schistosomiasis control in adults: a call for action towards the goal of universal health coverage. Eur. J. Pub. Health.

[91] Skivington K. (2021). Framework for the development and evaluation of complex interventions: gap analysis, workshop and consultation-informed update. Health Technol. Assess..

[92] Diaz A.V. (2023). Reaching the World Health Organization elimination targets for schistosomiasis: the importance of a one health perspective. Philos. Trans. R. Soc. Lond. Ser. B Biol. Sci..

[93] Freeman M.C. (2013). Integration of water, sanitation, and hygiene for the prevention and control of neglected tropical diseases: a rationale for inter-sectoral collaboration. PLoS Negl. Trop. Dis..

[94] Pannucci C.J., Wilkins E.G. (2010). Identifying and avoiding bias in research. Plast. Reconstr. Surg..

[95] Althubaiti A. (2016). Information bias in health research: definition, pitfalls, and adjustment methods. J. Multidiscip. Healthc..

[96] Lubanga A.F. (2024). Exploring the role of community involvement in reducing the burden of schistosomiasis and other neglected tropical diseases in Malawi: where are we in the fight against neglected tropical diseases?. Res. Rep. Trop Med..

[97] Zdesenko G. (2022). Pharmacogenetics of praziquantel metabolism: evaluating the cytochrome p450 genes of Zimbabwean patients during a schistosomiasis treatment. Front. Genet..

[98] Hanton A.J. (2022). Frequency distribution of cytokine and associated transcription factor single nucleotide polymorphisms in Zimbabweans: impact on schistosome infection and cytokine levels. PLoS Negl. Trop. Dis..

[99] Brooker S., Michael E. (2000). The potential of geographical information systems and remote sensing in the epidemiology and control of human helminth infections. Adv. Parasitol..

[100] Seto E.Y.W., Carlton E.J., Michael E., Spear R.C. (2010). Modelling Parasite Transmission and Control.

